# Solventless Synthesis of Poly(pyrazolyl)phenyl-methane Ligands and Thermal Transformation of Tris(3,5-dimethylpyrazol-1-yl)phenylmethane

**DOI:** 10.3390/molecules22030441

**Published:** 2017-03-11

**Authors:** Edith Rodríguez-Venegas, Efrén V. García-Báez, Francisco J. Martínez-Martínez, Alejandro Cruz, Itzia I. Padilla-Martínez

**Affiliations:** 1 Laboratorio de Química Supramolecular y Nanociencias, Instituto Politécnico Nacional-UPIBI, Av. Acueducto s/n Barrio la Laguna Ticomán, Ciudad de México C.P. 07340, Mexico; edith_rove@hotmail.com (E.R.-V.); efren1003@yahoo.com.mx (E.V.G.-B.); alcralmx@yahoo.com.mx (A.C.); 2 Facultad de Ciencias Químicas, Universidad de Colima, Km. 9 Carretera Colima-Coquimatlán, Coquimatlán C.P. 28400, Mexico; fjmartin@ucol.mx

**Keywords:** thermal synthesis, tris(pyrazolyl)phenylmethane, bis(pyrazolyl)phenylmethane, poly(pyrazolyl)methane, three-bladed propeller, X-ray molecular structure

## Abstract

The solventless synthesis of tris(pyrazolyl)phenylmethane ligands of formula C_6_H_5_C(Pz^R2^)_3_ (R = H, Me), starting from PhCCl_3_ and 3,5-dimethylpyrazole (Pz^Me2^) or pyrazole (Pz) was performed. The sterically crowded C_6_H_5_C(Pz^Me2^)_3_ is thermally transformed into the bis(pyrazolyl)(*p*-pyrazolyl)phenylmethane ligand Pz^Me2^-C_6_H_4_CH(Pz^Me2^)_2_. In this compound both Pz^Me2^ rings are linked through the N-atom to the methine C-atom. At higher temperatures, the binding mode of Pz^Me2^ changes from N1 to C4. All transformations occurred via quinonoid carbocation intermediates that undergo an aromatic electrophilic substitution on the 4-position of Pz^Me2^. Reaction conditions were established to obtain five tris(pyrazolyl)phenylmethane ligands in moderate to good yields. ^1^H- and ^13^C-NMR spectroscopy and X-ray diffraction of single crystals support the proposed structures.

## 1. Introduction

Poly(pyrazolyl)methane ligands RR’C(Pz)_2_, RC(Pz)_3_ and C(Pz)_4_ are the neutral analogues of the anionic poly(pyrazolyl)borates [1,2,3], where Pz can be either an unsubstituted or C-substituted pyrazolyl unit. They are formally derived by replacing the apical borate anionic moiety [BR]^−^ by the isoelectronic CR group. Trofimenko, in his pioneering work in the mid-1960s, called the poly(pyrazolyl)methane and poly(pyrazolyl)borate ligands scorpionates [4]. They are versatile hard N-donor ligands that can coordinate in a mono-, bi-, or tridentate fashion to a metal center [5]. The coordination, organometallic and related chemistry of tris(pyrazolyl)methane ligands have been reviewed elsewhere [6]. Bis(pyrazol-1-yl)methane ligands, [RR’C(Pz)_2_], have been used as synthetic precursors to a subclass of scorpionates called heteroscorpionates [7,8]. Their applications in the design and synthesis of metal complexes with a wide range of applications have been recently reviewed [8,9].

Steric and electronic effects modulate the chemical properties of the corresponding coordination and organometallic complexes of poly(pyrazol-1-yl)methane ligands. Small differences in topology, flexibility, donor properties, modes of coordination and decomposition pathways of these ligands, can lead to significantly different properties in their metal complexes.

Our investigations on iridium organometallic complexes formed using tris(pyrazol-1-yl)methane indicated that the steric effect exerted by this ligand strongly determines the fate of the organic moiety in the coordination sphere of the metal as well as the energetics of the olefinic C-H bond activation reactions [10,11,12].

The chemistry of poly(pyrazolyl)methanes has been relatively underdeveloped. However, this family of ligands can act as more than a simple spectator in the course of the chemical reactions due to the possibility of temporary changes in denticity and its ambidentate nature after the removal of the bridge CH proton [13]. Furthermore, the significant number of examples confirm that the properties and reactivity of the corresponding metal complexes differ from that of the tris(pyrazol-1-yl)borate analogues.

More recently, the use of poly(pyrazolyl)methanes in supramolecular chemistry has become more frequent. Bis(pyrazol-1-yl)(pyridin-x-yl)methane ligands give rise to a rich variety of complexes and supramolecular frameworks with a wide range of transition metals [14]. The crowded tris(pyrazolyl)methane chelate in a macrobicyclic structure was synthesized in order to create a cavity with well-defined dimensions and shape [15]. Also, aryl-*o*-substituted-bis(pyrazol-1-yl)methanes (RSPhCH(Pz^Me2^)_2_) have been synthesized to give rise to supramolecular microporous structures such as coordination polymers and macrocycles [16]. Other areas of development are catalysis, bioinorganic and biologically inspired chemistry [17]. In this field, the chemistry of Re and Tc complexes anchored by tris(pyrazolyl)methanes have potential relevance in the field of biomedical applications, particularly in radiopharmaceutical research [18].

In general, poly(pyrazolyl)methanes CH_2_(Pz)_2_, CH(Pz)_3_ and C(Pz)_4_ have been prepared by treating the pyrazolate salt with CH_2_Cl_2_, CHCl_3_ or CCl_4_ in the presence of phase transfer catalysts. This technique has been particularly exploited to prepare tris(pyrazol-1-yl)methanes of formula HC(Pz^R^)_3_ (R = 3,5-Me, 3-Ph, 3-*^i^*Pr, 3-*^t^*Bu, 3,5-*^i^*Pr) [19,20,21,22]. The metal catalyzed condensation of (Pz)_2_E = O (E = C, S) with aldehydes or ketones provides an efficient route to unsymmetrical tris(pyrazol-1-yl)methane ligands. However, similar chemistry is unsuccessful with substituted pyrazolyl rings [23]. The preparation of “mixed” tris(pyrazolyl)methanes with two or three different pyrazolyl rings (HCPz^R^(Pz^R′^)_2_ or HCPz^R^Pz^R′^Pz^R″^) has been achieved by the scrambling of tris(3,5-dimethylpyrazolyl)methane with substituted pyrazoles in acid media [24]. More recently, a third generation of scorpionates with a substituted-aryl ring bound to the carbon bridge of tris(pyrazol-1-yl)methane, has been synthesized starting from trifluoromethylaniline [25]. Methods for synthesizing bis and tris(pyrazol-1-yl)methanes have been recently reviewed [26].

**Figure 1:**
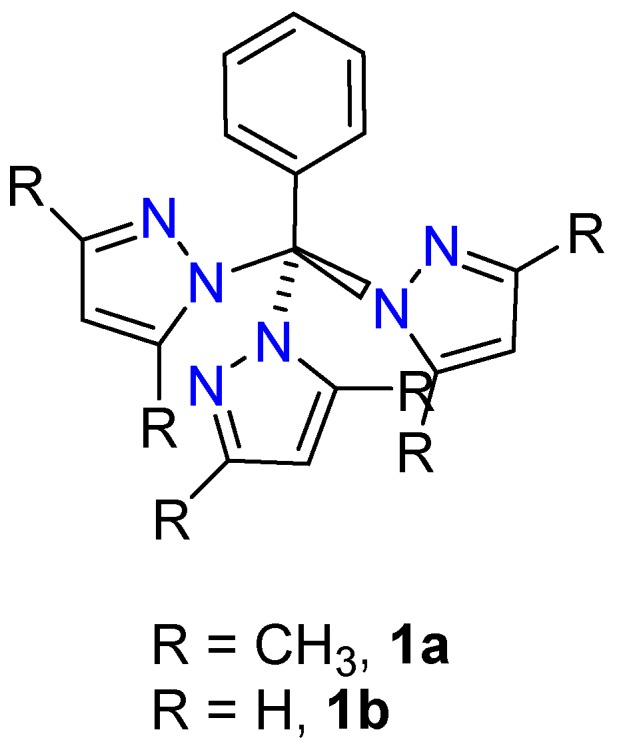
Target compounds C_6_H_5_C(Pz^Me2^)_3_ (**1a**) and C_6_H_5_C(Pz)_3_ (**1b**).

In an effort to contribute to the design of a new and improved synthesis of poly(pyrazol-1-yl)methane ligands, we decided to explore the synthesis without solvent (solventless synthesis) of tris(3,5-dimethylpyrazol-1-yl)phenylmethane (**1a**) and tris(pyrazol-1-yl) phenylmethane (**1b**), starting from α,α′,α″-trichlorotoluene (PhCCl_3_) and 3,5-dimethylpyrazole (Pz^Me2^) or pyrazole (Pz), (Figure 1). In this sense, thermal treatment [27] as well as sonochemical methods [28], mechanochemistry [29], microwave [30] and infrared [31] irradiation have been used for chemical synthesis driven by the search for new and cleaner synthetic methodologies. To the best of our knowledge, the sterically crowded compounds **1a** and **1b** have never been described before, whereas PhCCl_3_ has been scarcely used in spite of its similarity to CHCl_3_. It has been reported as the starting material for the synthesis of the hexadentate N6-donor phenyltris[3-(2-pyridyl)pyrazol-1-yl]methane [32].

## 2. Results and Discussion

### 2.1. Screening Reactions with Pz^Me2^

**Scheme 1:**
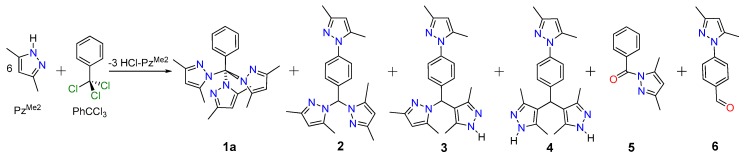
Thermal reaction of 3,5-dimethylpyrazol (Pz^Me2^) with α,α′,α″-trichlorotoluene (PhCCl_3_).

Since the reaction of pyrazoles with PhCCl_3_ has been reported to occur in low yields [32], we decided to explore the heating of 3,5-dimethylpyrazole (Pz^Me2^) without solvent while varying several different reaction conditions such as temperature, time, stoichiometry and the reaction atmosphere. Results are listed in Table 1 by numbered entries as yields of the isolated products after column chromatography. Initially, the reaction was performed in an open flask equipped with a condenser, the Pz^Me2^ to PhCCl_3_ ratio was chosen as 6:1, the excess of Pz^Me2^ was used to trap the evolved HCl, the mixture was melted (100 °C) and stirred for 10 h. A stronger base than Pz^Me2^ was not used to trap the evolved HCl in order to prevent the production of 1,1,2,2-tetrachloro-1,2-diphenylethane as side-product [33]. In the above conditions, compounds **2**, **5** and **6** were isolated in low yields from the reaction mixture (entry 1) and sublimated Pz^Me2^ was observed on the top of the flask (Scheme 1).

In order to avoid the sublimation of Pz^Me2^ and to improve yields, we used a sealed glass ampoule instead of a flask. The same quantities of reagents were used and heated at 120 °C for 24 h. In this case, the new product **4** was isolated in low yield (entry 2). The same reaction carried out at 80 °C for 48 h, leads to only small amounts of compound **5** (entry 3). These results suggest the transformation of the target compound **1a** into compounds **2** and **4**. However, as compound **1a** was not present in any of the last conditions, we carried out the reaction in two steps, the first at 80 °C and the second at 120 °C for 24 h each. In this case compounds **2** (53%) and the new compound **3** (27%) appeared as principal products (entry 4). We tried the same reaction lowering the initial reaction time to 24 h and the Pz^Me2^ to PhCCl_3_ ratio in 8:1 and 4:1 proportions, but the yields of compounds **2** and **3** were lower (entries 5 and 6, respectively). In all cases, compounds **5** and **6** appeared as oxidation products in the reaction mixture.

To avoid the formation of the oxidation products **5** and **6**, the reaction was carried out in a one-step reaction, with a 6:1 ratio of reactants, at 80 °C for 24 h in a sealed ampoule under vacuum. In this case, compound **1a** was obtained as the only product in 15% yield (entry 7). The best results to obtain compound **1a** in 30% yield were when the reaction was performed in the same reaction conditions but increasing the reaction time to 48 h (entry 8). Compound **2** was isolated in 70% yield in the two steps of temperature conditions at 80 °C and 120 °C for 48 h and 24 h, respectively (entry 9). Finally, compound **4** was isolated in 14% yield increasing the temperature to 160 °C for 72 h (entry 10).

It is worth mentioning that, to the best of our knowledge, compounds **1a**–**4** have not been reported before, compound **5** was reported in 1935 [34] and compound **6** is not known but the pyrazole analogue of **6** has been reported elsewhere [35].

### 2.2. Effect of Temperature and Time on the Composition of the Reaction Mixtures

In order to find the appropriate temperature that would favor one of the ligands **1a**–**4**, three reactions were performed using two temperature steps. The first step was fixed at 80 °C and the second at 100, 120 or 160 °C for 24 h each. In all cases, the reaction at 80 °C for 24 h was used as reference with 30% composition of compound **1a**. The composition of the reaction mixtures was quantified by HPLC and the results are summarized in Table 2. These experiments and those summarized in Table 3 were performed at a smaller scale (1/5) than the screening experiments summarized in Table 1; thus differences in the composition of the reaction mixtures are explained because of mass dependent conductive/convective heat transfer processes.

From the above results, it can be concluded that compounds **2** and **3** are formed at 100 and 120 °C, respectively, at the expense of compound **1a**. Compound **4** remained at 160 °C as the only product in low quantity. These results suggest that compound **1a** is successively transformed into compounds **2**–**4** with the increase in the temperature of reaction, accompanied by extensive decomposition at 160 °C. As expected, compounds **1a** and **2**, with C-N bonds, are produced at lower temperatures; on the contrary, compounds **3** and **4**, with C-C bonds, are favored at higher temperatures.

In an independent experiment, the temperature was fixed at 120 °C but the course of the reaction was monitored by HPLC for three days. The daily percent composition of the reaction mixture is listed in Table 3. A mixture of compounds **1a**–**4** was found the first day of reaction at 120 °C. In the next two days, the composition of the mixture showed an increase in compound **4** with the corresponding decrement of compounds **1a**–**3**. These results confirm that once formed, compounds **1**–**3** are consecutively transformed into compound **4**.

Compounds **2**–**4** contain a built-in entry point for further functionalization, and they could be used as monomers for new polyamines or ligands for the synthesis of MOF’s [36]. In addition, both nitrogen atoms of each pyrazole ring in compound **4** are available for further coordination. Suzuki coupling methodology starting from 1-trityl-1*H*-pyrazol-4-ylboronate pinacol esters and brominated aromatic precursors has been used to achieve this structural feature in the synthesis of oligo-(1*H*-pyrazol-4-yl)-arenes [37].

### 2.3. Reaction between PhCCl_3_ and Pz

**Scheme 2:**
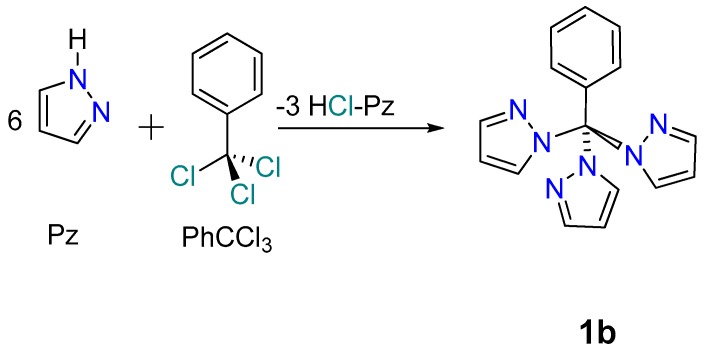
Thermal reaction of pyrazole with α,α′,α″-trichlorotoluene.

The reaction of pyrazole and PhCCl_3_ was performed using the reaction conditions described in entry 9 of Table 1. In these conditions, tris(pyrazolyl)phenylmethane **1b** was isolated in 50% yield as the only product, Scheme 2. Due to the absence of more products, no other reaction conditions were tested.

### 2.4. Stucture by NMR and IR

Compounds **1a** and **1b** possess *C3* symmetry in agreement with three pyrazole rings bonded to the bridge carbon atom of the former PhCCl_3_. This was confirmed by the ^1^H-NMR spectrum of **1a** that shows two signals for each of methyl groups (δ 2.18, 1.62). One of them appears shielded by 0.4 ppm compared with that of (Pz^Me2^)CH (δ 2.09, 2.03) [21]. The CH of three Pz^Me2^ rings appeared at δ 5.95. Compound **1b** shows the signals for Pz-ring protons at δ 7.73 (d), 7.52 (d) and 6.36 (t). The typical 1:2:2 pattern for a monosubstituted benzene ring is present in the ^1^H-NMR spectra of both compounds. Finally, the bridge carbon atom is at δ 94.3 and 94.4 in ^13^C-NMR for **1a** and **1b**, respectively.

The ^1^H-NMR spectrum of compound **2** shows two sets of signals at δ 2.28, 2.27 and 2.26, 2.20 ppm in a 2:1 proportion corresponding to the methyl groups of the Pz^Me2^ rings. Two pyrazole CH are at δ 5.86 and 5.98 ppm in agreement with a molecule with *C2v* symmetry. The methine proton and carbon atoms appear as a singlet at δ 7.65 (^1^H) and δ 73.5 (^13^C), very similar to those values reported for (Pz^Me2^)_2_CHPh [26]. In addition, the presence of signals at δ 7.40 and 6.98, as doublets, are in agreement with the typical pattern for a *p*-substituted benzene ring. They were assigned to the *m*- and *o*-protons, respectively, by NOE effect when irradiating the bridge methine CH. This pattern is also observed for compounds **3** and **4** with similar chemical shifts.

The symmetry is lost in compound **3** because the Pz^Me2^ rings are bonded differently to the bridge methine carbon atom. The C-N bonded Pz^Me2^ ring, shows signals at δ 5.86 and 105.8 for H4 and C4, respectively. No signal corresponding to H4 was observed and C4 of Pz^Me2^ ring appeared at δ 113.6 as singlet in the ^13^C{^1^H} NMR spectrum of the C-C bonded ring. In addition, a broad singlet at δ 144.0, corresponding to the CMe carbon atom of the two tautomers in equilibrium, was observed; the respective NH appeared at δ 7.60. Also, a singlet appeared for each remaining four methyl carbon atoms. It is worth noting that the chemical shifts of the methine proton and carbon atoms are at δ 6.45 and 57.0, respectively a value in the middle of the corresponding values for compounds **2** and **4**.

In compound **4**, both Pz^Me2^ rings are linked by a C-C bond to the bridge methine carbon atom. Some relevant changes in both the ^1^H- and ^13^C-NMR spectra are appreciated in agreement with its *C2v* symmetry. The CH of the pyrazole ring is absent in the ^1^H-NMR spectrum, a broad signal at δ 12 for the NH proton appears instead, as well as a singlet at δ 115.7 in the ^13^C{^1^H} NMR spectrum, corresponding to C4 of both Pz^Me2^ rings. Tautomeric equilibrium, typical of NH pyrazole heterocycles, is present leading to a broad signal for both CMe carbon atoms of each Pz^Me2^ at δ 142.7. Finally, the bridge methine proton and carbon atoms are shifted to high fields (δ 5.3, 39.3).

The ^1^H-NMR spectrum of compound **5** shows the characteristics set of signals corresponding to a monosubstituted benzene ring as well as a singlet at δ 6.06 and two signals in δ 2.63 and 2.25 for two methyl groups of the Pz^Me2^ ring. The signal at δ 168.7 in the ^13^C-NMR spectrum was assigned to a C=O group which was confirmed by the presence of an IR stretching band at 1694 cm^−1^. These spectroscopic data are in agreement with an amide group. The NMR of compound **6** shows the characteristic signal for an aldehyde: a singlet at δ 10.04 in the ^1^H-NMR spectrum, and at δ 191.4 in the ^13^C-NMR one. The stretching frequency at 1697 cm^−1^ confirmed the presence of the C=O group. The signals in the ^1^H-NMR spectrum are consistent with the presence of a *p*-substituted benzene ring at δ 7.97 and 7.67 and, three singlets at δ 6.06, 2.41 and 2.31 for the Pz^Me2^ ring.

### 2.5. Proposed Reaction Mechanism

**Scheme 3:**
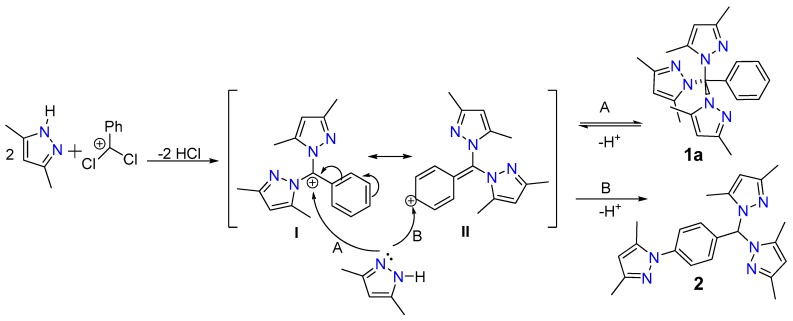
Proposed mechanistic pathway for the reaction of Pz^Me2^ and PhCCl_3_.

Based on the above results a mechanistic pathway is proposed for the heating reaction of Pz^Me2^ and PhCCl_3_. A nucleophilic attack of two Pz^Me2^ molecules to carbocation PhCCl_2_^+^ formed from PhCCl_3_, give the intermediate carbocation **I**. The fate of carbocation **I** depends on temperature, at 80 °C, the attack of the third Pz^Me2^ leads to compound **1a** (Scheme 3, path A). Compound **1a** reversibly releases one pyrazole ring to regenerate carbocation **I** when temperature is increased to 100 °C. Then, compound **2** is obtained by the attack of the third Pz^Me2^ to the resonance quinonoid aryl carbocation intermediate **II** (Scheme 3, path B) loosing of one proton to recover the aromatic form. The intrinsic instability of compound **1a** could be attributed to the steric repulsion exerted by both phenyl and methyl groups, which favors the breaking of the bridge C-N bond to liberate the steric constraint. α-chlorobenzyl cations have been reported as intermediates in reactions of hydrolysis [38] whereas radicals or cation radicals are produced only when initiators are added [39].

**Scheme 4:**
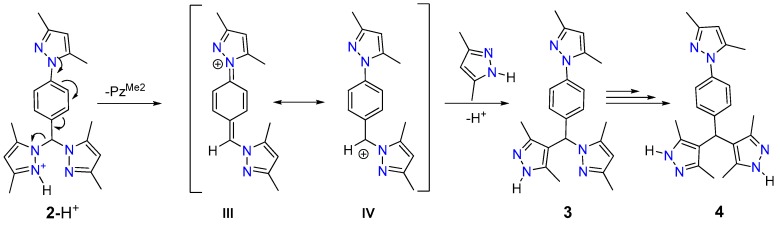
Proposed mechanistic transformation of compound **2** to **3** and **4**.

The synergy of both steric and electronic effects is evident in the reaction of the unsubstituted pyrazole (Pz) and PhCCl_3_ that produce the stabile compound **1b** as the only product. No decomposition or transformation of **1b** was observed at the temperature of 120 °C. Pyrazole is non-steric constrained and less basic than Pz^Me2^ (p*K*_a_ values are 2.85 and 14.2 for Pz, 4.28 and 15.1 for Pz^Me2^) [40,41]. Compounds **3** and **4** are produced from compound **2**. Their formation implies the substitution of N-bonded Pz^Me2^ by C-bonded Pz^Me2^. At 120 °C, compound **2** irreversibly releases one protonated pyrazole ring. This step requires the participation of an electron donating group in the aryl ring to weaken the C-N bond via the formation of the quinonoid carbocation intermediate **III**, which is stabilized by resonance. The intermediate resonance hybrid form **IV** performs an aromatic electrophilic substitution (AES) on the 4-position of Pz^Me2^ to form compound **3**. The subsequent elimination-substitution of the second pyrazole leads to the formation of compound **4** (Scheme 4). The activating role of the electron donating group in the aryl ring have been demonstrated to be necessary in an elimination-addition mechanism that provides access to tris(pyrazolyl)-toluidines [25]. On the other hand, AES reaction in Pz^Me2^ is reported to occur under mild conditions [42].

### 2.6. Single Crystal Structures of ***1a***–***b*** and ***2***

**Figure 2:**
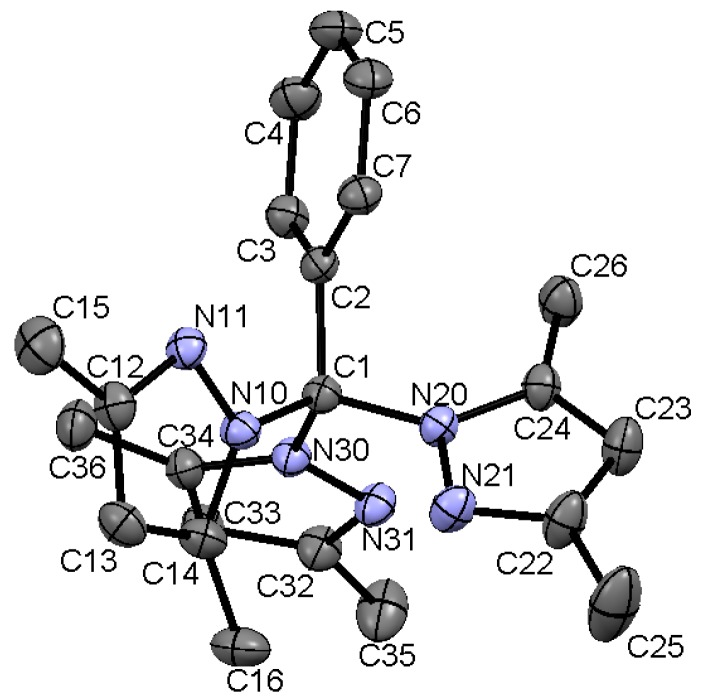
ORTEP perspective of compound **1a** at 30% probability level, hydrogen atoms are omitted for clarity. Selected bonds lengths (Å) and angles (°): N10-C1 1.4657(19), N20-C1 1.4725(18), N30-C1 1.479(2), C1-C2 1.532(2), N30-C1-C2 111.47(11), N20-C1-C2 107.84(11), N30-C1-C2 111.75(11), N10-C1-N20 109.86(11), N10-C1-N30 106.48(12), N20-C1-N30 109.43(11), N11-N10-C1 119.04(11), N21-N20-C1 118.72(11), N31-N30-C1 119.76(11).

The structures of compounds **1a** and **1b** were unambiguously confirmed by single-crystal X-ray diffraction analysis. Suitable crystals of compound **1a** were obtained from hexane solution. Compound **1a** crystallized in the monoclinic system with a space group P21/c. The molecular structure is displayed in Figure 2.

The geometry around the C1 bridge carbon atom is nearly tetrahedral with an average N-C-N bond angles of 109(2)° and the N-C-C_Ph_ angle of 110(2)°. The mean C-N and C-C_Ph_ bond distances of 1.477(7) Å and 1.532(2) Å, respectively, are in close correspondence with single bonds (1.493(20), 1.513(14) Å) [43]. The three Pz^Me2^ rings adopt a three-bladed propeller structure around the axial phenyl with R^3^-C-N-N torsion angles of ±8.97(17)°, ±101.46(15)° and ±141.34(13)°. Thus, racemic helical molecules of **1a** are found to exist as both left-(Λ) and right-handed (Δ)-enantiomers in the asymmetric unit of the crystal lattice. Among several possible conformers, compound **1a** adopts a *sp*, *ac*, *ac* conformation (synperiplanar (*sp*), synclinal (*sc*), antiperiplanar (*ap*) and anticlinal (*ac*)).

**Figure 3:**
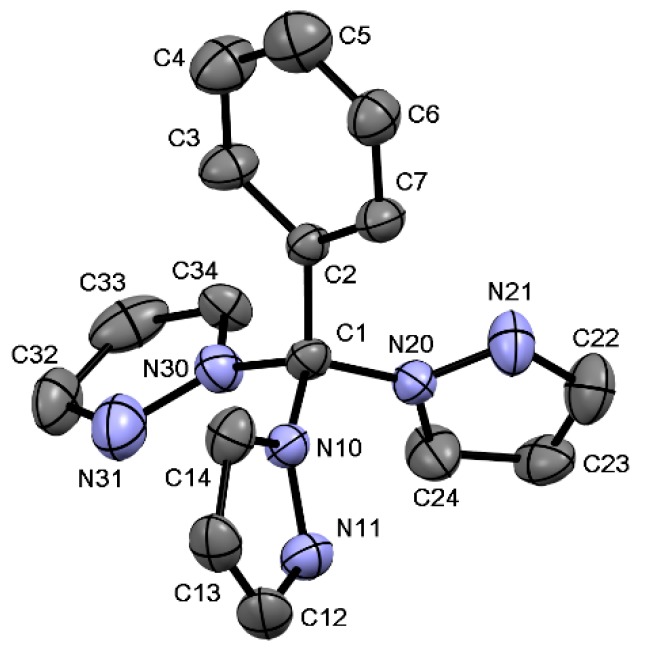
ORTEP perspective of compound **1b** at 25% probability level; hydrogen atoms are omitted for clarity. Selected bonds lengths (Å) and angles (°): N10-C1 1.472(5), N20-C1 1.452(5), N30-C1 1.477(5), C1-C2 1.525(6), N30-C1-C2 111.8(3), N10-C1-N20 109.9(3), N10-C1-N30 107.8(3), N11-N10-C1 119.1(3), N21-N20-C1 120.8(4), N31-N30-C1 126.2(4)

Suitable crystals of compound **1b** were obtained from a saturated solution in AcOEt. Crystals of **1b** crystallize in the orthorhombic system in the acentric *P2_1_2_1_2_1_* space group. The molecular structure is displayed in Figure 3. Bond distances and angles are very similar to those found in compound **1a** (average values): N-C-N bond angle of 110(2)°, N-C-C_Ph_ angle of 111(2)° and C-N and C-C_Ph_ bond distances of 1.476(16) Å and 1.525(5) Å, respectively. Three blades are formed by Pz rings; the values of the R^3^-C-N-N torsion angles are ±87.17(4)° (*sc*), ±104.2(4)° (*ac*) and ±166.6(4)° (*ap*).

Since the structures of several ligands **7**–**15**, similar to compounds **1a** and **1b**, have been reported [24,25,44,45,46,47,48,49,50], a brief comparison between them seems to be appropriate. The structures, R^3^-C-N-N torsion angles values and the conformation of pyrazole rings in compounds **1a**,**b** and **7**–**15** are listed in Table 4.

All these molecules are very similar; their bond distances and angles have no evident differences regardless of the steric demand from the substituents in the pyrazole ring or in the apical carbon atom. However, steric effects seem to determine the conformation adopted by pyrazole rings in the three-bladed propeller structure. Compounds **1a** and **7**, the most crowded, are in such disposition that one pyrazole is in *sp* and the other two are in *ac* conformations (*sp* + 2*ac*). As long as steric demand is diminished, one ring adopts an *ap* conformation and the other two are concomitantly twisted to finally adopt the *sp* conformation (2*sp* + *ap*) in the less crowded compounds **14** and **15**. As expected, substituents in the pyrazole rings exert more steric demand than those located in the apical carbon atom.

**Figure 4:**
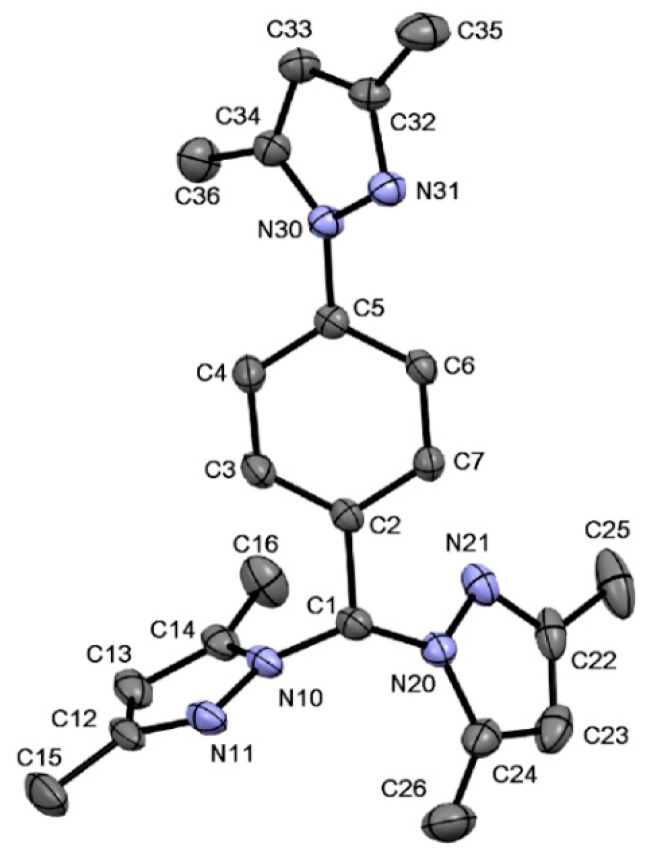
ORTEP perspective of compound **2** at 25% probability level, hydrogen atoms and the molecule of water are omitted for clarity. Selected bonds lengths (Å) and angles (°): N10-C1 1.464(5), N20-C1 1.445(5), C1-C2 1.521(5), N10-C1-C2 112.5(3), N20-C1-C2 114.1(3), N10-C1-N20 111.5(3), N11-N10-C1 116.7(3), N21-N20-C1 121.4(3).

Compound **2** crystalized with 0.5 molecules of water in the asymmetric unit as a monoclinic system with a space group C2/c. The crystal structure is shown in Figure 4. Bond distances and angles are very similar to those found in compound **1a** (average values): N-C-N bond angle of 111.5(3)°, N-C-C_Ph_ angle of 113.3(4)° and C-N and C-C_Ph_ bond distances of 1.455(7) Å and 1.521(5) Å, respectively. Two blades are formed by Pz^Me2^ rings, the values of the R^3^-C-N-N torsion angles are −105.0(3)° and −29.2(5)° corresponding to *ac*, s*p* conformation, respectively.

**Figure 5:**
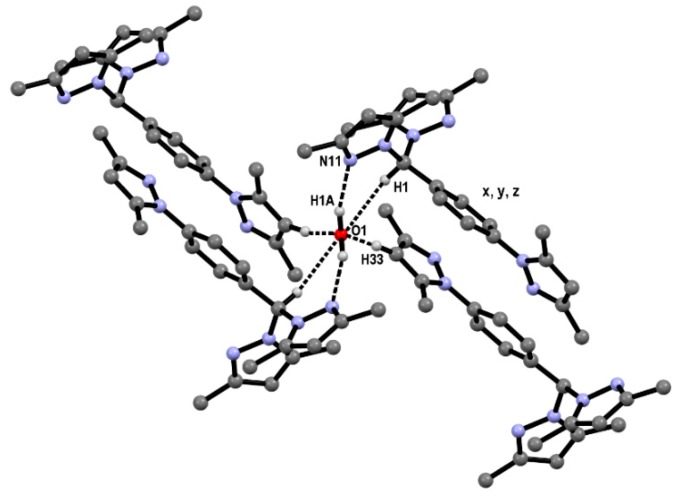
Supramolecular structure of compound **2**. Geometry of hydrogen bonding: H1A∙∙∙N11 = 1.96(6) Å, O1∙∙∙N11 = 2.925(4) Å, O1-H1A∙∙∙N11 = 156(6)° and H1∙∙∙O1 = 2.51 Å, C1∙∙∙O1 = 3.441(6) Å, C1-H1∙∙∙O1 = 137° (symmetry code = −1/2 + x, ½ + y, z); H33∙∙∙O1 = 2.51 Å, C33∙∙∙O1 = 3.410(6) Å, C33-H33∙∙∙O1 = 158° (symmetry code = x, −1 + y, z).

Two molecules of **2** are hydrogen bonded to one molecule of water. One of them forms a pseudo six membered ring *R*^2^_2_(6) [51] through strong O-H∙∙∙N interactions, with the participation of pyrazole N atom as the acceptor and by soft C(*sp*^3^)-H∙∙∙O interactions. The other molecule of **2** is linked to the oxygen atom of the water molecule by pyrazole C4-H as donor (Figure 5). This supramolecular arrangement highlights the acid-base sites in the molecule, in agreement with the proposed mechanism of transformation of compound **2** into compounds **3** and **4** (*vide supra*).

## 3. Materials and Methods

### 3.1. Chemicals

Pyrazole, 3,5-dimethylpyrazole, α,α′,α″-trichlorotoluene and solvents were reagent grade and used as received.

### 3.2. Instrumental Methods

Melting points were measured on an IA 9100 apparatus (Electrothermal, Staffordshire, UK) and are uncorrected. IR spectra were recorded using a 3100 FT-IR Excalibur Series spectrophotometer (Varian, Randolph, MA, USA) equipped with an ATR system. Mass spectra were obtained in a 3900-GC/MS system (Varian, Palo Alto, CA, USA) with an electron ionization mode. Elemental analyses (EA) were performed on a 2400 elemental analyzer (Perkin-Elmer, Waltham MA, USA). ^1^H- and ^13^C-NMR spectra were recorded on a Varian Mercury 300 (^1^H, 300.08; ^13^C, 75.46 MHz) instrument in CDCl_3_ solutions for compounds **1**–**6**, and in DMSO-d6 solutions for compound **7**, SiMe_4_ as the internal reference was used. Chemical shifts are in ppm and *^n^J*(H-H) in hertz. The chemical shift assignments were performed on the basis of ^1^H- and ^13^C-NMR NOE, COSY and HETCOR experiments. Composition of the reaction mixtures were performed in a Varian 9010 HPLC instrument at 252 nm. A C18 column of 5 μm particle size and 25 cm length and 0.5 cm wide was used. Elution was achieved with MeOH/H_2_O mixtures in a 7:3 proportion, unless otherwise specified.

### 3.3. X-ray Diffraction Methods

Single-crystal X-ray diffraction data of molecules **1a**, **1b** and **2** were recorded on a D8 Quest CMOS (Bruker, Karlsruhe, Germany) or Nonius Kappa (Rotterdam, The Netherlands) area detector diffractometers with Mo K α radiation, λ = 0.71073 Å. A table listing the crystallographic data is provided as Supplementary Material. The structures were solved by direct methods using SHELXS97 [52] program of WinGX package [53]. The final refinement was performed by full-matrix least-squares methods on F2 with SHELXL97 [52] program. H atoms on C were geometrically positioned and treated as riding atoms, with C-H = 0.93–0.98 Å, and with Uiso(H) = 1.2Ueq(C). The hydrogen atoms of the water molecule were found by Fourier difference and freely refined. The program Mercury was used for visualization, molecular graphics and analysis of crystal structures [54]. The software used to prepare material for publication was PLATON [55]. Crystallographic data for the structures in this paper have been deposited with the Cambridge Crystallographic Data Centre as supplementary publication CCDC numbers 1527853 (**1a**), 1527852 (**1b**) and 1527854 (**2**). Copies of the data can be obtained free of charge on application to CCDC, 12 Union Road, Cambridge CB2 1EZ, UK, (Fax: +44-01223-336033 or E-Mail: deposit@ccdc.cam.ac.uk). Crystals suitable for X-ray analysis were obtained from the saturated hexane solution of compound **1a** and from DMSO-H_2_O solution (1:1) of compound **1b**. Compound **2** slowly crystalized with one molecule of water in the asymmetric unit from the semi-solid remaining after solvent evaporation.

*Crystal Data for C_22_H_26_N_6_* (**1a**, M = 374.5 g/mol): monoclinic, space group P21/c (No. 14), *a* = 10.0680(3) Å, *b* = 14.6640(3) Å, *c* = 15.3674(5) Å, β = 114.204(3)°, V = 2069.35(31) Å^3^, Z = 4, T = 298(2) K, Dcalc = 1.200 g/cm^3^, 23332 reflections measured (3.4° ≤ 2Θ ≤ 27.5°), 4632 unique (Rint = 0.046, Rsigma = 0.048) which were used in all calculations. The final R1 was 0.053 (I > 2σ(I)) and wR2 was 0.125 (all data).

*Crystal Data for C_16_H_14_N_6_* (**1b**, M = 290.33 g/mol): orthorhombic, space group P2_1_2_1_2_1_ (No. 19), *a* = 7.0198(8) Å, *b* = 14.2693(16) Å, *c* = 14.6994(17) Å, α = β = γ = 90°, V = 1449.37(3) Å^3^, Z = 4, T = 293(2) K, Dcalc = 1.331 g/cm^3^, 8182 reflections measured (2.0° ≤ 2Θ ≤ 24.0°), 2280 unique (Rint = 0.08, Rsigma = 0.139) which were used in all calculations. The final R1 was 0.055 (I > 2σ(I)) and wR2 was 0.1203 (all data).

*Crystal Data for C22H26N6∙0.5H_2_O* (**2**, M = 383.49 g/mol): monoclinic, space group C 2/c (No. 15), *a* = 20.9571(15) Å, *b* = 9.5425(6) Å, *c* = 21.8515(16) Å, β = 103.528(2)°, V = 4248.69(22) Å^3^, Z = 4, T = 100(2) K, Dcalc = 1.200 g/cm^3^, 19257 reflections measured (2.4° ≤ 2Θ ≤ 25.0°), 3584 unique (Rint = 0.043, Rsigma = 0.0351) which were used in all calculations. The final R1 was 0.089 (I > 2σ(I)) and wR2 was 0.188 (all data).

### 3.4. Synthetic Procedures

#### 3.4.1. Open Flask Procedure

3,5-Dimethylpyrazole (Pz^Me2^, 5.00 g, 52.0 mmol) was placed in a round bottom flask, submerged in a silicon oil bath and heated at 110 °C with constant stirring. When the pyrazole was molten, α,α′,α″-trichlorotoluene (PhCCl_3_, 246 µL) was added dropwise every hour until complete 1.230 mL (8.660 mmol) had been added. Heating was maintained for 5 h to obtain a yellow viscous liquid. The products were purified by column chromatography on silica gel using a 6:4 mixture of hexane/EtOAc as eluent (Table 1, entry 1).

#### 3.4.2. Sealed Ampoule Procedure

Pz^Me2^ (5.00 g, 52.0 mmol) was ground in a mortar and placed into a 10 mL glass ampoule with PhCCl_3_ (0.93 mL for 8:1; 1.23 mL for 6:1 or 1.85 mL for 4:1 ratio). The ampoules were sealed at room temperature, introduced into a convection oven and allowed to react at the temperature and time indicated in Table 1. The obtained viscous brown colored mixtures were dissolved in 50 mL of CHCl_3_ to remove them from the ampoules and subjected to column chromatography on silica gel. Compounds **1**–**3** and **5**–**6** were purified using hexane/EtOAc mixtures from 8:2 to 6:4 proportions as eluents.

#### 3.4.3. Vacuum Sealed Ampoule Procedure

Pz^Me2^ (5.00 g, 52.0 mmol) was ground in a mortar and placed into a 10 mL glass ampoule with PhCCl_3_ (1.23 mL, 8.66 mmol, 6:1). The ampoules were frozen at −30 °C, sealed under vacuum and then introduced into a convection oven and allowed to react at the temperature and time indicated in Table 1. The reaction mixtures were treated as indicated in Section 3.4.2.

### 3.5. Synthesis of Compounds ***1***–***6***

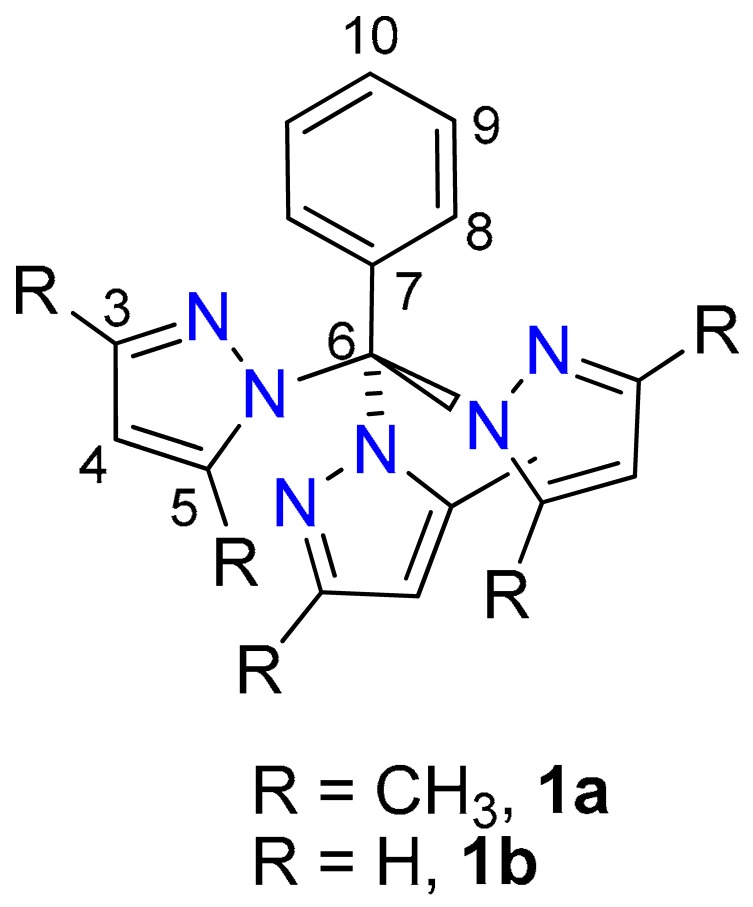



*1,1′,1″-(Phenylmethanetriyl)tris(3,5-dimethyl-1H-pyrazole) (***1a***)*. This compound was synthesized following the procedure described in Section 3.4.3 heating at 80 °C for 24 h (Table 1, entry 8) to obtain 0.975 g (2.60 mmol, 30% yield) of a white crystalline solid m.p. = 90 °C after column chromatography. ^1^H-NMR (CDCl_3_): δ 7.39 (t, 1H, *^3^J* = 6.9, H-10), 7.32 (t, 2H, *^3^J* = 7.0, H-9), 7.02 (d, 2H, *^3^J* = 7.0, H-8), 5.95 (s, 3H, H-4), 2.18, 1.62 (s, 9H each, 6CH_3_). ^13^C-NMR (CDCl_3_): δ 146.9 (C-3), 143.9 (C-5), 138.1 (C-7), 130.5 (C-8), 129.6 (C-9), 127.3 (C-10), 109.1 (C-4), 94.3 (C-6), 14.3, 12.9 (CH_3_). IR neat (cm^−1^): 1697 (C=C), 1562 (C_ar_), 1450, 1411, 1374, 1316, 1027, 890, 716. EA % found [calculated for C_22_H_26_N_6_ (374.49 g·mol^−1^)]: 70.06 (70.56, C), 7.11 (7.00, H), 22.01 (22.40, N).

*1,1′,1″-(Phenylmethanetriyl)tris(1H-pyrazole) (***1b***)*. A crystalline solid m.p. = 105 °C was obtained (1.95 g, 55% yield), starting from 4.97 g (17.16 mmol) of pyrazole and 1.7 mL (12.2 mmol) of PhCCl_3_. Procedure 3.4.3 was followed using two temperature steps: 80 °C and 120 °C for 48 and 24 h each, respectively (Table 1, entry 9). The crude product was solubilized in EtOAct and filtered, whereupon compound **1b** crystallized from the solution. ^1^H-NMR (CDCl_3_): 7.73 (d, 3H, H-3), 7.52 (d, 3H, *^4^J* = 2.6, H-5), 7.48 (t, 1H, *^3^J* = 7, H-10), 7.41 (t, 2H, *^3^J* = 8.7, H-9), 7.09 (d, 2H, *^3^J* = 8, H-8), 6.36 (t, 3H, *^4^J* = 2.6, 1.7, H-4). ^13^C-NMR (CDCl_3_): 141.6 (C-3), 137.6 (C-7), 132.6 (C-5), 130.8 (C-10), 128.9 (C-9), 128.5 (C-8), 106.7 (C-4), 93.4 (C-6). IR (neat, ν/cm^−1^): 1682 (C=N), 1451, 1419, 1388, 1196, 1095, 1087, 864, 746 (Pz out of plane). EA % found [calculated for C_16_H_14_N_6_ (290.32 g·mol^−1^)]: 66.16 (66.19, C), 4.91 (4.86, H), 28.91 (28.95, N).

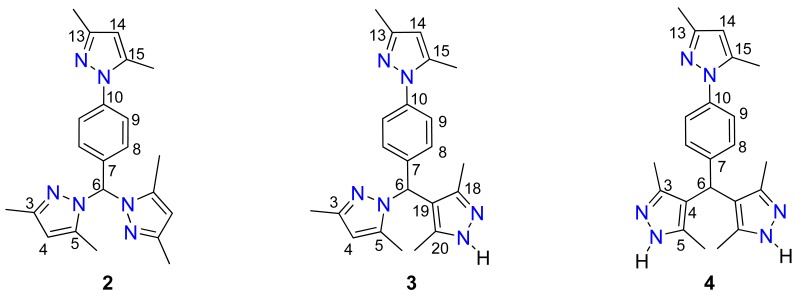



*1,1′-((4-(3,5-Dimethyl-1H-pyrazol-1-yl)phenyl)methylene)bis(3,5-dimethyl-1H-pyrazole) (***2***)*. Synthesized following the procedure described in Section 3.4.3 using two temperature steps at 80 °C and 120 °C for 48 and 24 h each, respectively (Table 1, entry 9). After column chromatography, 2.28 g (6.09 mmol, 70% yield) of compound **2** as a viscous yellow liquid which slowly solidified after standing as a yellow solid m.p. = 97 °C was obtained. ^1^H-NMR: δ 7.65 (s, 1H, H-6), 7.40 (d, 2H, *^3^J* = 8.8, H-9), 6.98 (d, 2H, *^3^J* = 8.8, H-8), 5.98 (s, 1H, H-14), 5.86 (s, 2H, H-4), 2.28, 2.27 (s, 6H each, 4CH_3_), 2.26, 2.20 (s, 3H each, 2CH_3_). ^13^C-NMR: δ 149.5 (C-13), 148.8 (2C-3), 141.3 (2C-5), 140.1 (C-15), 139.7 (C-10), 135.8 (C-7), 127.9 (2C-8), 124.6 (2C-9), 107.5 (C-14), 107.2 (2C-4), 73.5 (C-6), 13.9, 13.0 (4CH_3_), 13.7, 12.7 (2CH_3_). IR (neat, ν/cm^−1^): 1612 (C=N), 1556 (C=C), 1520 (C_ar_), 1417 (CH_3_), 750 (pyrazole out of plane). MS (%): 374.0 (M+, 3), 279.0 (100), 183.9 (5), 162.0 (26), 94.8 (12.5). EA % found [calculated for C_22_H_26_N_6_ (374.48 g·mol^−1^)]: 70.50 (70.56, C), 7.17 (7.00, H), 21.70 (22.44, N).

*1,4′-((4-(3,5-Dimethyl-1H-pyrazol-1-yl)phenyl)methylene)bis(3,5-dimethyl-1H-pyrazole) (***3***)*. Synthesized following the procedure described in Section 3.4.2 using two temperature steps of 80 °C and 120 °C for 24 h each (Table 1, entry 4) to obtain, after column chromatography, 0.88 g (2.35 mmol, 27% yield) of a yellow viscous liquid that after a while turned into a beige solid, m.p. = 126 °C. ^1^H-NMR: δ 7.60 (b, 1H, NH), 7.31 (d, 2H, *^3^J* = 8.5, H-9), 6.91 (d, 2H, *^3^J* = 8.5, H-8), 6.45 (s, 1H, H-6), 5.96 (s, 1H, H-14), 5.86 (s, 1H, H-4), 2.27, 2.25, 2.20, 2.18 (s, 3H each, 4CH_3_), 1.92 (s, 6H, 2CH_3_). ^13^C-NMR: δ 149.2 (C-13), 147.9 (C-3), 144.0 (C-18,20), 140.0 (C-15), 139.7 (C-5), 139.6 (C-10), 139.0 (C-7), 127.9 (2C-8), 124.8 (2C-9), 113.6 (C-19), 107.2 (C-14), 105.8 (C-4), 57.0 (C-6), 14.0, 13.7, 12.7, 11.5 (4CH_3_), 11.8 (2CH_3_). IR (neat, ν/cm^−1^): 1613, 1589 (C=N), 1554 (C=C), 1519 (Car), 1425 (CH_3_), 749 (pyrazole out of plane). MS (%): 374.0 (M+, 3), 279.0 (100), 94.8 (67.5). EA % found [calculated for C_22_H_26_N_6_ (374.48 g·mol^−1^)]: 70.20 (70.56, C), 7.10 (7.00, H), 21.82 (22.40, N).

*4,4′-((4-(3,5-Dimethyl-1H-pyrazol-1-yl)phenyl)methylene)bis(3,5-dimethyl-1H-pyrazole) (***4***)*. Synthesized following the procedure described in Section 3.4.3 after heating at 160 °C for 72 h (Table 1, entry 10). Excess Pz^Me2^ was sublimed and the remaining solid was stirred twice in 25 mL of 10% NaHCO_3_ solution, decanted, washed twice with 50 mL of water, filtered and air dried. After column chromatography, 0.460 g (1.23 mmol, 14% yield) of a pale beige solid which decomposes at 249 °C was isolated. ^1^H-NMR: δ 12.0 (b, 2H, NH), 7.38 (d, 2H, *^3^J* = 8.0, H-9), 7.13 (d, 2H, *^3^J* = 7.4, H-8), 6.03 (s, 1H, H-14), 5.3 (s, 1H, H-6), 2.24, 2.15 (s, 3H each, 2CH_3_), 1.72 (b, 12H, 4CH_3_). ^13^C-NMR: δ 148.3 (C-13), 142.7 (b, 2C-5 and 2C-3), 142.6 (C-7), 139.7 (C-15), 138.2 (C-10), 129.6 (2C-8), 124.5 (2C-9), 115.7 (2C-4), 107.6 (C-14), 39.3 (C-6), 14.0, 12.8 (2CH_3_), 11 (b, 4CH_3_). IR (neat, ν/cm^−1^): X (C=N), 1558 (C=C), 1517 (C_ar_), 1413 (CH_3_), 760 (pyrazole out of plane). EA % found [calculated for C_22_H_26_N_6_ (374.48 g·mol^−1^)]: 69.41 (70.56, C), 7.03 (7.00, H), 21.52 (22.40, N).

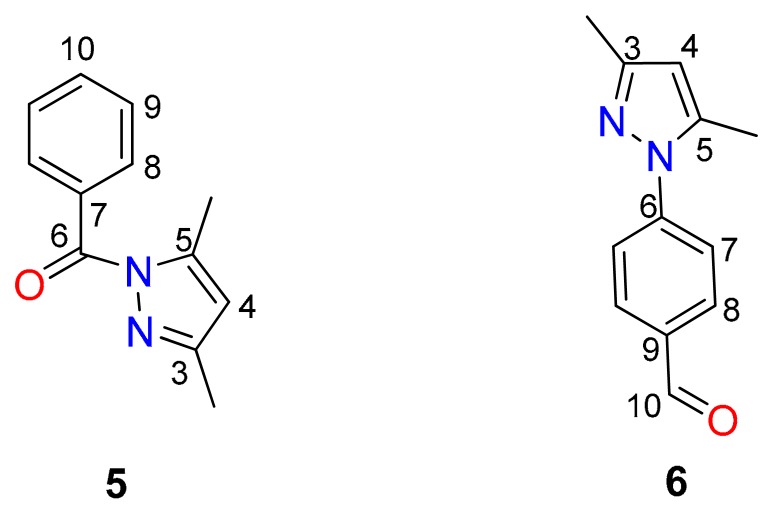



*(3,5-Dimethyl-1H-pyrazol-1-yl)(phenyl)methanone (***5***)*. Synthesized following the procedure 3.4.1 to afford 0.173 g (6%) of a yellow viscous liquid, after column chromatography. ^1^H-NMR: δ 8.00 (d, 2H, *^3^J* = 8.0, H-8), 7.56 (t, 1H, *^3^J* = 7.5, H-10), 7.46 (t, 2H, *^3^J* = 7.4, H-9), 6.06 (s, 1H, H-4), 2.63, 2.25 (s, 3H each, 2CH_3_). ^13^C-NMR: δ 168.7 (C-7), 152.4 (C-3), 145.3 (C-5), 132.7 (C-10), 131.6 (C-8), 128.1 (C-9), 111.4 (C-4), 14.6, 14.1 (2CH_3_). IR (neat, ν/cm^−1^): 1694 (C=O), 1583 (C=C), 1448 (CH_3_). MS (%): 200.9 (M+, 7.5), 172.0 (11.3), 104.9 (100), 76.9 (45.0), 51.0 (20).

*4-(3,5-Dimethyl-1H-pyrazol-1-yl)benzaldehyde (***6***)*. Synthesized following procedure 3.4.1 to afford 0.343 g (10%) of a yellow viscous liquid after column chromatography. ^1^H-NMR: δ 10.04 (s, 1H, CHO), 7.97 (d, 2H, *^3^J* = 8.34, H-8), 7.67 (d, 2H, *^3^J* = 8.1, H-7), 6.06 (s, 1H, H-4), 2.41, 2.31 (s, 3H each, 2CH_3_). ^13^C-NMR: δ 191.4 (C-10), 150.5 (C-9), 145.0 (C-5), 140.0 (C-6), 134.5 (C-3) 130.9 (C-8), 124.1 (C-7), 108.9 (C-4), 13.8, 13.2 (2CH_3_). IR (neat, ν/cm^−1^): 1697 (C=O), 1555 (C=C), 1514 (Car), 1418 (CH_3_). MS (%): 200.3 (M+, 27.5), 199.9 (100), 170.9 (11.3), 129.9 (17.5), 76.9 (20).

### 3.6. Temperature Effects Experiments 

Four ampoules containing 1.000 g of Pz^Me2^ (10.4 mmol), previously ground in a mortar, and 0.250 mL of PhCCl_3_ (1.7 mmol) each were cooled at −30 °C and sealed under vacuum. The reference ampoule was introduced into a preheated oven at 80 °C and heated at the same temperature for 24 h. The other three ampoules were heated in two steps of temperature at 80/100, 80/120 and 80/160 °C for 24 h at each temperature. The obtained viscous reaction mixtures were dissolved in 30 mL of CHCl_3_ to homogenize and the solvent was evaporated to dryness, except the last ampoule whose content was homogenized by grinding it in a mortar. Samples of 10 mg/mL in methanol as solvent were prepared and then diluted to achieve 2.5 mg/mL for HPLC measurements. Chromatograms were recorded with 10 µL of each sample eluted in the same conditions as the calibration curve. Calibration curves were obtained from methanol solutions of 2.0, 1.5, 1.0 and 0.5 mg/mL of compounds **1a**–**4** each.

### 3.7. Time Effect Experiments

Three ampoules were prepared as described for temperature effects but starting with 5.00 g of Pz^Me2^ (52.0 mmol) and 1.23 mL of PhCCl_3_ (8.66 mmol) each, and heated in an oven at 120 °C for one, two and three days. The samples for HPLC were prepared as before.

## 4. Conclusions

In summary, the solventless synthesis of tris(pyrazolyl)phenylmethane ligands C_6_H_5_C(Pz^R2^)_3_ (R = H, Me), starting from PhCCl_3_ and 3,5-dimethylpyrazole (Pz^Me2^) or pyrazole (Pz) was achieved. The sterically crowded C_6_H_5_C(Pz^Me2^)_3_ is labile due to both steric and electronic reasons; therefore it is thermally transformed at 120 °C into bis(pyrazolyl)(*p*-pyrazolyl)phenylmethane ligand, Pz^Me2^-C_6_H_4_CH(Pz^Me2^)_2_. In this compound both Pz^Me2^ rings are linked through the N-atom to the methine-bridge C-atom. At longer times of reaction or at temperatures beyond 120 °C, the binding mode of Pz^Me2^ changes from N1 to C4. Reaction conditions were established to obtain compound **1a** in 30%, **1b** in 55%, **2** in 70%, **3** in 27% and **4** in 14% yields. The structures were proposed on the basis of ^1^H- and ^13^C-NMR spectroscopy and single crystal X-ray diffraction. The three Pz^Me2^ rings, in compounds **1a** and **1b**, adopt a three-bladed propeller structure around the axial phenyl ring. Comparison to the structures of other tris(pyrazolyl)phenylmethane ligands allowed to conclude that steric effects determine the conformation adopted by pyrazole rings: (*sp* + 2*ac*) in the most crowded and (2*sp* + *ap*) in the less crowded compounds. Substituents in the pyrazole rings exert more steric demand than those located in the apical carbon atom.

## Figures and Tables

**Table 1:** Reaction conditions of heating 3,5-dimethylpyrazol with α,α′,α″-trichlorotoluene.

Entry	Vessel	Ratio ^1^	Temperature (°C)/Time (h)		Products Isolated Yields (%)					
			Step 1	Step 2	1a	2	3	4	5	6
1	Open flask	6:1	100/10	none	---	11	---	---	6	10
2	SA ^2^	6:1	120/24	none	---	---	---	8	Trace	Trace
3	SA	6:1	80/48	none	---	---	---	---	7	Trace
4	SA	6:1	80/24	120/24	---	53	27	---	6	Trace
5	SA	8:1	80/24	120/24	---	48	25	---	8	Trace
6	SA	4:1	80/24	120/24	---	27	7	---	9	Trace
7	VSA ^3^	6:1	80/24	none	15	---	---	---	---	---
8	VSA	6:1	80/48	none	30	---	---	---	---	---
9	VSA	6:1	80/48	120/24	---	70	2	---	---	Trace
10	VSA	6:1	160/72		---	---	---	14	---	---

^1^ Pz^Me2^ to PhCCl_3_; ^2^ Sealed ampoule; ^3^ Vacuum sealed ampoule.

**Table 2:** Composition of the reaction mixture (%) after two heating steps at three temperatures.

Comp.	80 °C to T_2_ (°C)		
	100	120	160
**1a**	26	18	0
**2**	11	55	0
**3**	0	12	0
**4**	0	0	11

**Table 3:** Composition of the reaction mixture (%) after heating at 120 °C.

Comp.	Heating Time (Days)		
	1	2	3
**1a**	13	8	0
**2**	7	0	0
**3**	5	3	0
**4**	23	35	40

**Table 4:** Torsion angles and conformation of three bladed propeller structures of general formula (Pz^R1R2^)_3_C^R3^.

	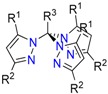		
Comp.	Substituents	Torsion Angle (°) R^3^CNN	Pz^R1R2^ Conformation ^1^
**1a**	R^1^ = R^2^ = CH_3_, R^3^ = C_6_H_5_	−8.97(17), 101.46(15), 141.34(13)	*sp*, *ac*, *ac*
**7** [44]	R^1^ = CH_3_, R^2^ = C_6_H_5_, R^3^ = H	−18.3, 133.4, 148.6	*sp*, *ac*, *ac*
**8** ^2^ [45,46]	R^1^ = R^2^ = CH_3_, R^3^ = H	(±)25(5), (±)116(5), (±)152(5)	*sp*, *ac*, *ap*
**1b**	R^1^ = R^2^ = H, R^3^ = C_6_H_5_	−87.2(4), −104.2(4), −166.6(4)	*sc*, *ac*, *ap*
**9** [25]	R^1^ = R^2^ = H, R^3^ = *o*-C_6_H_4_-NH_2_	29.89(13), 87.54(11), 179.85(9)	*sp*, *sc*, *ap*
**10** [25]	R^1^ = R^2^ = H, R^3^ = *p*-C_6_H_4_-NH_2_	−25(11), −83(14), 173(7)	*sp*, *sc*, *ap*
**11** [47]	R^1^ = R^2^ = H, R^3^ = *p*-C_6_H_4_-C_6_O_2_S	42.6(7), 73.6(8), 166.4(2)	*sc*, *sc*, *ap*
**12** [48]	R^1^ = R^2^ = H, R^3^ = *p*-C_6_H_4_OH	−45.02(14), −68.86(14), 173.04(14)	*sc*, *sc*, *ap*
**13** [49]	R^1^ = R^2^ = H, R^3^ = CH_2_OH	−34.49(13), −62.50(12), 174.56(9)	*sc*, *sc*, *ap*
**14** [24]	R^1^ = H, R^2^ = CH_3_, R^3^ = H	24.48, 27.97, 170.87	*sp*, *sp*, *ap*
**15** [50]	R^1^ = R^2^ = R^3^ = H	13.6, 15.7, 176.0	*sp*, *sp*, *ap*

^1^ Torsion angle from 0° to ±30° is called synperiplanar (*sp*), ±30° to ±90° synclinal (*sc*), ±90° to ±150° anticlinal (*ac*) and ±150° to ±180° antiperiplanar (*ap*). ^2^ Average value of two molecules in the asymmetric unit.

## Supplementary Materials

Supplementary materials are available online.
