# Supplementary material for: Solventless Synthesis of Poly(pyrazolyl)phenyl-methane Ligands and Thermal Transformation of Tris(3,5-dimethylpyrazol-1-yl)phenylmethane
Source: Molecules. 2017 Mar 11;22(3):441. doi: 10.3390/molecules22030441 (PMC6155200; doi:10.3390/molecules22030441)
Supplement: Supplementary file 1 [file molecules-22-00441-s001.docx]

*Supplementary material for the article*

**Solventless synthesis of poly(pyrazolyl)phenylmethane ligands and thermal transformation of tris(3,5-dimethylpyrazol-1-yl)phenylmethane**

**Edith Rodríguez-Venegas ^1^, Efrén V. García-Báez ^1^, Francisco J. Martínez-Martínez ^2^, Alejandro Cruz ^1^ and Itzia I. Padilla-Martínez ^1,^***

^1^ Laboratorio de Química Supramolecular y Nanociencias, Instituto Politécnico Nacional-UPIBI, Av. Acueducto s/n Barrio la Laguna Ticomán, Ciudad de México, C.P. 07340, Mexico; ipadillamar@ipn.mx.

^2^ Facultad de Ciencias Químicas, Universidad de Colima, Km. 9 Carretera Colima-Coquimatlán, C.P. 28400, Coquimatlán, Colima, Mexico; fjmartin@ucol.mx

***** Correspondence: ipadillamar@ipn.mx; Tel.: +(52)555-729-6000

**Table S1.** Complete crystallographic data for compounds **1a**, **1b** and **2**.

| **Crystal Data** | **1a** | **1b** | **2** |
| --- | --- | --- | --- |
| Formula | C_22_ H_26_ N_6_ | C_16_H_14_N_6_ | C_22_ H_26_ N_6_ 0.5H_2_O |
| Formula Weight | 374.5 | 290.33 | 383.49 |
| Crystal System | Monoclinic | Orthorhombic | Monoclinic |
| Space group | P2_1_/c (No. 14) | P2_1_2_1_2_1_ (No. 19) | C2/c (No. 15) |
| a[Å]  b[Å]  c[Å]  α, β, γ(deg) | 10.0680(3)  14.6640(3)  15.3674(5)  90.00, 114.204(3), 90.00 | 7.0198(8)  14.2693(16)  14.6994(17)  90.00 | 20.9571(15)  9.5425(6)  21.8515(16)  90.00, 103.528(2), 90.00 |
| V [Å^3^] | 2069.35(31) | 1449.37(3) | 4248.69(22) |
| Z | 4 | 4 | 4 |
| D(calc) [g/cm^3^] | 1.200 | 1.331 | 1.200 |
| μ(MoKα) [mm^-1^] | 2.503 | 0.086 | 0.076 |
| F(000) | 800 | 776 | 1640 |
| Crystal Size [mm] | 0.40 x 0.30 x 0.30 | 0.40 x 0.35 x 0.30 | 0.40 x 0.33 x 0.30 |
| Data Collection |  |  |  |
| θ Min-max [Deg] | 3.4, 27.5 | 2.0, 24.0 | 2.4, 25.0 |
| Dataset | -12:12;-18:17 ; -19:19 | -7:8;-16:16 ; -16:13 | -25:25 ;-11:11 ; -26:26 |
| Tot., Uniq. Data, R(int) | 23332, 4632, 0.046 | 8182, 2280, 0.08 | 19257, 3584, 0.043 |
| Observed Data [I > 2.0 sσ(I)] | 3512 | 1128 | 3260 |
| Refinement |  |  |  |
| Nref, Npar | 4632, 259 | 2200, 200 | 3584, 263 |
| R, w_R2_, S | 0.053, 0.125, 1.026 | 0.055, 0.1203, 0.753 | 0.089, 0.188, 1.252 |
| Max. and Av. Shift/Error | 0.010, 0.00 | 0.00, 0.00 | 0.00, 0.00 |
| Min. and Max. Resd. Dens. [e/ Å^3^] | -195, 0.228 | -0.224, 0.190 | -0.231, 0.331 |
